# Editorial Notes

**Published:** 1914-06

**Authors:** 


### Editorial Notes.

**Geheimrat Professor Killian at the Royal Infirmary.**

Suspension laryngoscopy marks a new era in laryngology and a further development of the methods of direct laryngoscopy, tracheoscopy and bronchoscopy which we owe to the genius of Killian, and which opened up regions for treatment that heretofore had been practically inaccessible. Inspection of growths, ulcers, and other pathological conditions and their treatment, and also the removal of foreign bodies even from the bronchi of the second division, have become almost familiar and commonplace in special throat clinics by means of Killian's tubes, or the improved patterns of Brünings and Chevalier Jackson. But the use of these tubes involves monoptic vision, and the engagement of one hand in holding the instrument, and therefore in laryngeal conditions, at any rate, suspension laryngoscopy—which exposes to view the larynx and trachea by using the weight of the patient's head to retract the base of the tongue and epiglottis by means of a specially constructed spatula, and thus leaves both the operator's hands free, and at the same time gives an uninterrupted view of the whole larynx, and of the pharynx and hypo-pharynx—is, from a practical standpoint, the opening up of a new field in laryngology. Professor Killian was appointed to the Semon Lectureship at the London University this year, taking as the subject his method of suspension laryngoscopy. It was a privilege to have a demonstration of the latest developments of this method at the Bristol Royal Infirmary, although suspension laryngoscopy had already been employed there for the removal of growths, etc. A number of the Honorary

Medical Staffs of the Royal Infirmary and General Hospital, as well as the Vice-Chancellor of the University of Bristol and laryngologists from Bath and Exeter, received their distinguished colleague in the throat department of the Royal Infirmary. It is interesting to hear from Prof. Killian how he came to think of the method, for it was due to his requiring an artist to make coloured drawings of the larynx, etc., as seen through the direct laryngoscopic lamp in the cadaver. He found that it was too trying to support the lamp and tube *in situ* for a sufficient length of time for the artist to work, and therefore supported the larynx with a spatula, by means of a hook depending from a clamp on the dissecting room table, the head of the subject hanging in the supine position over the end of the table. Then it was that the new and more complete view of the regions of the larynx appeared to him so much more perfect than that obtained in the usual way through the tube spatula, that it occurred to the Professor that the same enlarged view would be equally well obtained in the living patient. Once the possibilities that lay in such a method had occurred to him, Professor Killian worked incessantly to devise and to improve the technique, and the instruments that had to be constructed, with the result that he has given to laryngology another epoch-making advance.

Although we were thus enabled to profit by the presence of the inventor to witness suspension laryngoscopy under the best auspices, the primary object of his visit to the Royal Infirmary was to see for himself the newer methods there employed for operating intranasally for frontal sinusitis; and after witnessing this operation on a case with left frontal sinus and double antral suppuration, he inspected a number of others in whom the same operation had previously been done. As the most widely-practised external operation for frontal sinus suppuration is that introduced by Professor

Killian some years ago, he was particularly interested in the newer intranasal methods.

**Winsley Sanatorium for Consumption.**

THE Ninth Annual Meeting of this Institution was held on May the ninth, at the Sanatorium, the President, Dr. Shingleton Smith, in the chair. We cannot congratulate the Board of Management on the way they are conducting the medical side of the work, that is the most essential and important side, and when the President felt called upon to make a public criticism of his managing board, it may be assumed firstly that the grounds for complaint are exceptionally strong and well-founded, and secondly that the criticisms uttered were the least he could say consistent with the duty his high and responsible position imposes, and that on a less public occasion he might have said a good deal more. As it is, we note that the Annual Report contains no report from the Medical Consultative Board! The reason is that no report was invited, and no one who is conversant with the way the Medical Consultative Board's previous reports and recommendations have been treated by the lay committee could wonder if the medical men who constitute the Medical Consultative Board do not care to "turn the other cheek." When we recall the fact that the movement for the better control and treatment of the poor consumptive was initiated by medical men in Bristol and the Counties of Gloucester, Wilts and Somerset, that the first official committee was organised by the Bristol Medico-Chirurgical Society, that their efforts culminated in the building and equipping of the Sanatorium, that they directed the work in its inception, and that they taught the various lay bodies and Municipal Corporations how this Sanatorium should be worked, it is a matter for surprise that the lay members of the Committee should feel that they can ignore the medical guidance of their own Medical Board in medical matters. The Winsley Sanatorium Management quite recently had to face the resignation of a capable Resident Medical Officer, on the ground that it was difficult to carry on the medical work of the institution satisfactorily; firstly, because he was hampered in the control of the Institution, and secondly because the number of patients and the inadequacy of trained assistants rendered impossible that individual care and attention which is the essence of modern sanatorium methods.

Hitherto we find that one resident medical officer has had something like one hundred patients to supervise and to treat, and that the position accorded to him is by no means that of appertaining to a medical superintendent. The members of the House Committee and Board of Management deserve thanks for devoting much time and energy to the interests of the institution, and it is perhaps too much to expect the layman to realise fully how the morale of the institution depends on the personal factor of the medical superintendent, or how much this is as an essential element in the successful treatment as are the purely therapeutic methods employed. As it is, the Winsley Sanatorium partakes more of the character of an expensive convalescent home than an up-to-date scientific sanatorium for consumption.

We note that on the occasion of this annual meeting, as on previous occasions, a representative of the Bristol Corporation makes much of the fact that the Sanatorium had a large debt when it was built and equipped, and that the various public bodies which secured and paid for beds had discharged the debt. On the other hand, it may be pointed out that the institution could have been sold, and the financial soundness of the venture proved, had not the original committee preferred the sensible course of inducing the Municipal Corporations to meet their responsibilities to the poor consumptive of the cities by finding beds for them in a well-equipped sanatorium, and no doubt the representatives of the ratepayers were well assured that the money they thus invested was being well spent. As it is, the Corporations have obtained control of over twenty thousand pounds sunk in the institution by private donors, and the representatives of those donors have been so reduced that they have no effective voice whatever in the control of the institution that the private donors initiated and equipped. The ratepayers, represented by the Corporation, can hardly be indifferent to any waste of public money from lack of reasonable efficiency, and the State, from which now a large proportion of the funds for maintenance are derived, is not likely to acquiesce in the expenditure of enormous sums from the public purse unless the management places itself beyond the criticism of independent authority, much less its own Medical President, whose name has been so long associated with the treatment of consumption, or of its Medical Consultative Board, which comprises experts in tuberculosis. Now that the State provides such a large proportion of the cost of maintenance, it is to be hoped that the Local Government Board and the Charity Commissioners will see to it that the work is of the highest order, so that the results of the Winsley Sanatorium may be accepted as an indication of what may be accomplished by sanatorium methods properly carried out.

